# 900. Switching to DTG/3TC Fixed-Dose Combination (FDC) Is Non-inferior to Continuing a TAF-Based Regimen (TBR) in Maintaining Virologic Suppression Through 144 Weeks (TANGO Study)

**DOI:** 10.1093/ofid/ofab466.1095

**Published:** 2021-12-04

**Authors:** Olayemi Osiyemi, Faïza Ajana, Fiona Bisshop, Stéphane De Wit, Joaquín Portilla, Jean-Pierre Routy, Christoph Wyen, Mounir Ait-Khaled, Keith Pappa, Ruolan Wang, Peter Leone, Jonathan Wright, Brian Wynne, Jean A van Wyk, Michael Aboud, Kimberly Smith

**Affiliations:** 1 Triple O Research Institute PA, West Palm Beach, Florida; 2 Centre Hospitalier de Tourcoing, Tourcoing, Nord-Pas-de-Calais, France; 3 Holdsworth House Medical Brisbane, Brisbane, Queensland, Australia; 4 CHU St-Pierre, Brussels, Brussels Hoofdstedelijk Gewest, Belgium; 5 Hospital General Universitario de Alicante, Alicante, Comunidad Valenciana, Spain; 6 McGill University Health Center, Montreal, Quebec, Canada; 7 Medical practice Ebertplatz, Cologne, Germany, Cologne, Nordrhein-Westfalen, Germany; 8 ViiV Healthcare, London, England, United Kingdom; 9 GlaxoSmithKline, Stockley Park, England, United Kingdom

## Abstract

**Background:**

DTG/3TC is a complete 2-drug regimen (2DR) for the treatment of HIV-1 infection. Non-inferior virologic efficacy has been proven over 3 years in treatment-naive people living with HIV (PLWH) and 2 years in a stable switch setting.

**Methods:**

TANGO, a randomized, open-label, non-inferiority study, evaluates efficacy and safety of switching to DTG/3TC in PLWH who are virologically suppressed ( > 6 months, no prior virologic failure [VF], no major NRTI/INSTI resistance) vs remaining on a 3- or 4-drug TAF-based regimen (TBR), stratified by baseline 3rd agent class. Week 144 analyses assessed non-inferiority (NI) with a 4% NI margin for Snapshot virologic failure (VF) and 8% for virologic success (VS; FDA Snapshot algorithm, intention-to-treat–exposed [ITT-E] population).

**Results:**

Of 741 randomized/exposed pts (DTG/3TC: 369; TBR: 372), most pts entered the study on EVG/c (66%). For Week 144 Snapshot VF, switching to DTG/3TC was non-inferior to continuing TBR in the ITT-E analysis: 0.3% vs 1.3%; adjusted difference (95% CI): −1.1% (−2.4%, 0.2%) and superior to TBR in the per-protocol analysis: 0% vs 1.1%; adjusted difference: −1.1% (−2.3, −0.0); *P*=0.044 (2-sided). Snapshot VS was high in both arms and demonstrated non-inferiority (Table). Zero pts on DTG/3TC and 3 (0.8%) on TBR met confirmed virologic withdrawal criteria with no resistance observed. Zero pts on DTG/3TC and 6 (1.6%) on TBR discontinued for lack of efficacy. Overall AE rates were similar between arms (Table). TC, LDL-C, and triglycerides improved with DTG/3TC, HDL-C improved with TBR, with no difference in TC/HDL-C ratio between arms. Changes in eGFR (cystatin C) and proximal tubular function marker were similar across arms. Adjusted mean change from BL in weight was 2.2 and 1.7 kg in the DTG/3TC and TBR arms, respectively, and proportion of pts with > 10% weight increase was similar across arms (13% and 12%, respectively).

Table. Efficacy and Key Safety Results for the ITT-E and Safety Population

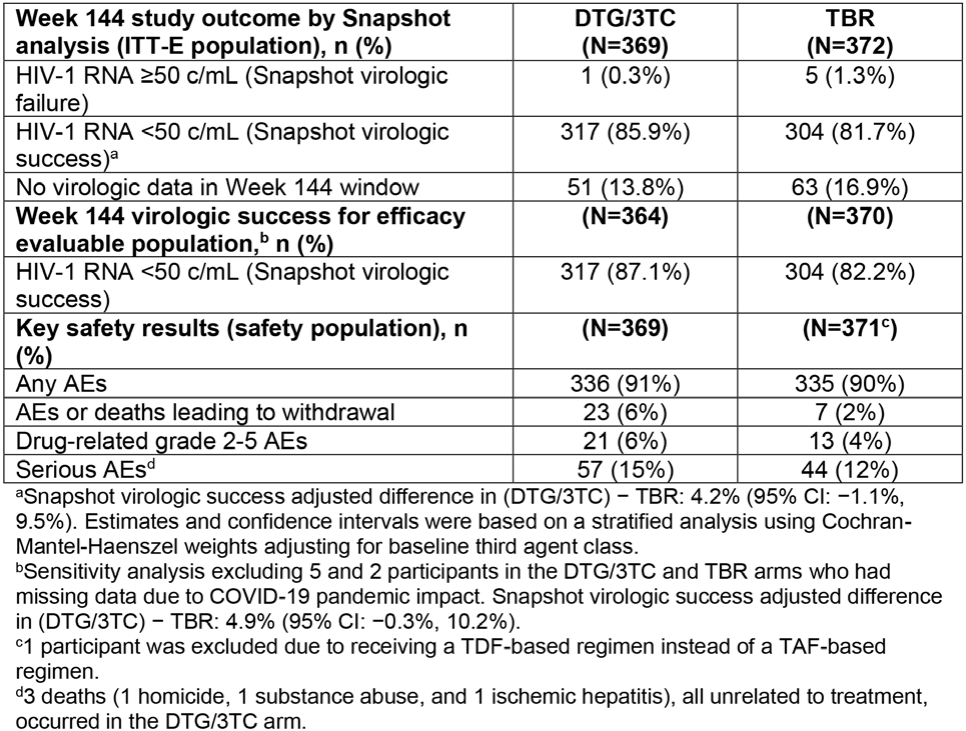

**Conclusion:**

Switching to the 2-drug regimen of DTG/3TC from a TAF-based 3- or 4-drug regimen resulted in high, non-inferior efficacy with zero confirmed virologic withdrawals and good tolerability over 3 years of treatment. DTG/3TC 2DR is a robust switch option with durable efficacy, good safety and tolerability, and a high barrier to resistance.

**Disclosures:**

**Olayemi Osiyemi, M.D**, **Gilead** (Advisor or Review Panel member, Speaker’s Bureau)**Merck** (Advisor or Review Panel member)**ViiV Healthcare** (Advisor or Review Panel member, Speaker’s Bureau) **Fiona Bisshop, MBBBS**, **Gilead** (Grant/Research Support)**ViiV Healthcare** (Grant/Research Support) **Stéphane De Wit, MD**, **Gilead** (Grant/Research Support)**Janssen** (Grant/Research Support)**Merck Sharpe & Dohme** (Grant/Research Support)**ViiV Healthcare** (Grant/Research Support) **Joaquín Portilla, MD**, **AbbVie** (Other Financial or Material Support)**Gilead** (Grant/Research Support, Other Financial or Material Support)**Janssen** (Grant/Research Support, Other Financial or Material Support)**Merck Sharpe & Dohme** (Other Financial or Material Support)**ViiV Healthcare** (Grant/Research Support, Other Financial or Material Support) **Jean-Pierre Routy, MD, FRCPC**, **ViiV Healthcare** (Grant/Research Support) **Mounir Ait-Khaled, PhD**, **ViiV Healthcare** (Employee) **Keith Pappa, PharmD**, **Glaxo Smith Kline** (Shareholder)**ViiV Healthcare** (Employee) **Ruolan Wang, Master of Science**, **ViiV Healthcare** (Employee) **Peter Leone, MD**, **viiv healthcare** (Employee) **Jonathan Wright, MSc**, **GlaxoSmithKline** (Employee, Shareholder) **Brian Wynne, MD**, **ViiV Healthcare** (Employee, Shareholder, I have shares in GSK, the part owner of ViiV) **Jean A. van Wyk, MB,ChB**, **GlaxoSmithKline** (Shareholder)**ViiV Healthcare** (Employee) **Michael Aboud, MBChB, MRCP**, **GlaxoSmithKline** (Shareholder)**ViiV Healthcare** (Employee) **Kimberly Smith, MD**, **GlaxoSmithKline** (Shareholder)**ViiV Healthcare** (Employee)

